# Interviews for the assessment of long-term incapacity for work: a study on adherence to protocols and principles

**DOI:** 10.1186/1471-2458-9-169

**Published:** 2009-06-02

**Authors:** Wout EL de Boer, Haije Wind, Frank JH van Dijk, Han HBM Willems

**Affiliations:** 1 Department of Quality of Life, TNO, Hoofddorp, The Netherlands; 2 Academic Medical Centre, University of Amsterdam, Coronel Institute of Occupational Health, Amsterdam, The Netherlands

## Abstract

**Background:**

Assessments for long-term incapacity for work are performed by Social Insurance Physicians (SIPs) who rely on interviews with claimants as an important part of the process. These interviews are susceptible to bias. In the Netherlands three protocols have been developed to conduct these interviews. These protocols are expert- and practice-based. We studied to what extent these protocols are adhered to by practitioners.

**Methods:**

We compared the protocols with one another and with the ICF and the biopsychosocial approach. The protocols describe semi-structured interviews with comparable but not identical topics. All protocols prescribe that the client's opinion on his capacity for work, and his arguments, need to be determined and assessed. We developed a questionnaire to elicit the adherence SIPs have to the protocols, their underlying principles and topics. We conducted a survey among one hundred fifty-five experienced SIPs in the Netherlands.

**Results:**

Ninety-eight SIPs responded (64%). All respondents used some form of protocol, either one of the published protocols or their own mix. We found no significant relation between training and the use of a particular protocol. Ninety percent use a semi-structured interview. Ninety-five percent recognise having to verify what the claimant says and eighty-three percent feel the need to establish a good relation (p = 0.019). Twelve topics are basically always addressed by over eighty percent of the respondents. The claimant's opinion of being fit for his own work or other work, and his claim of incapacity and his health arguments for that claim, reach a hundred percent. Description of claimants' previous work reaches ninety-nine percent.

**Conclusion:**

Our study shows professional consensus among experienced Dutch SIPs about the principle of assessment on arguments, the principle of conducting a semi-structured interview and the most crucial interview topics. This consensus can be used to further develop a protocol for interviewing in the assessment of incapacity for work in social insurance. Such a protocol can improve the quality of the assessments in terms of transparency and reproducibility, as well as by enabling clients to better prepare themselves for the assessments.

## Background

People at work get sick every now and then, generally for a short time. A minority of these remains sick for a longer time and some are forced to turn to social insurance. Arrangements for people with long-term incapacity for work exist in social insurance in many countries, among which the Netherlands and the UK. In these schemes, a benefit is possible for those insured that meet the legal criterion of being permanently unable to gain sufficient income because of illness or handicap [1]. This meets the requirements of what Gordon [2] called the 'handicapped role', or 'disability role' according to Waddell and Aylward [3]. That concept describes the health condition of the person as 'disabled', his rights to be (partly) exempt from work, his obligation to look for cure and rehabilitation, and his obligation to look for work that may still be fit for him. The legal criteria are formulated in abstract terms, which facilitate tailor-made assessments of people in very different circumstances [4]. In order to be granted a benefit, insured people have to file a claim and they have to be assessed. These assessments lead to conclusions about the residual capacity for work of the claimant in terms of the scheme of disability benefit. Between countries there is considerable variation in social insurance schemes, but the assessments of long-term incapacity for work are most often performed by specialised social insurance physicians (SIPs) [5]. This is, for example, the case in the Netherlands and the UK. The quality of these assessments is unknown. One might consider the degree of work resumption to be an indicator of the quality of the assessments as they predict the claimant's capacity for work. However, work resumption alone is not a valid quality indicator as it is influenced by the personal factors of the claimant (e.g. motivation, attitudes and beliefs, social factors) and by factors on the labour market. Relating incapacity for work only to objective medical findings would do an injustice to claimants as (in-) capacity for work is a relational concept that requires the consideration of work factors as well [3]. In several countries process indicators and expert based guidelines have been developed to support the work of the SIPs [6,7]. In one type of guideline, the profession of the SIPs makes clear how they consider that assessments should be done according to diagnostic categories [6,8]. In another type of guideline, prescriptions are provided about how to perform the assessments in general [6,9,10].

SIPs may use a number of sources to acquire information for their assessments. The first source is the claimant, who has knowledge of his situation and needs to have the opportunity to explain his claim and his arguments, and so to put forward grounds on which his claim is to be evaluated. Interviews are, therefore, crucial and they can be organised either face to face with the SIP (as is the case in the Netherlands and in the UK) or through an intermediate professional such as a medical specialist (e.g. Germany: [11]) or a social insurance officer (e.g. Sweden: [12]). Apart from the claimant, the SIP may also request information from the treating physician, the employer and external medical experts. Social insurance physicians in the Netherlands mainly base their judgement about the work ability of claimants on the information they receive from the claimants [13]. One might argue that the claimant's opinion of what he can and cannot do in work should be sufficient evidence on which to provide a benefit [14-16]. The claimant's opinion, however, may be governed by coping problems and economical interests and so the claimant may be biased [17,18]. Furthermore, the legal criteria for benefit for long-term incapacity for work are abstract [4] and it is unsure if claimants have a good understanding of the assessment criteria. So the interviews with the claimants are not only meant to be used for listening, but also to inform the claimant and to verify the claim against the legal criteria [19].

It is unknown how these interviews are conducted in practice. Guidelines for assessment of incapacity for work indicate what needs to be addressed in the interview. They do not indicate how this needs to be done – whether it is in a free conversation, following a form or using some structure. It seems, therefore, likely that every SIP develops his own routine, guided by his education, his experience and his preferences. This is not without risks: several studies show substantial differences in results between assessors, which underlines that these interviews do not meet criteria of reliability [20-22]. Both for society and for claimants, it is hard to accept that the final outcome of an assessment is not only depending of the physical or mental condition, but also on the person who performs the assessment.

A protocol that describes how to conduct a reliable interview to assess incapacity for work would be of value for both SIPs and claimants. Structured interviews are known to enhance the reliability of information gathering and conclusion [23]. In the Netherlands, three interview protocols have been drafted from practice to be used in the assessments. Based on these protocols, the profession has the opportunity to develop standards of good social insurance medicine. These protocols and their underlying principles provide an opportunity to study the professional consensus about these interviews. SIPs in the Netherlands receive, depending on where they work and get their education, training in one or several of these protocols, and they are free to use them or to adapt them to the SIPs' own wishes. This situation provided an opportunity to find out if there is professional consensus in practice on how to conduct the interviews. For this reason, we were looking for an answer to the following question:

*To what extent are SIPs familiar with the protocols and to what extent do they adhere to the principles of the protocols*?

## Methods

### Design

The design of the study is a descriptive survey among social insurance physicians.

### Participants and recruitment procedures

A total of one hundred fifty-five social insurance physicians (SIPs) were sent a questionnaire. These SIPs were selected from the nine hundred members of the Dutch Association of Insurance Medicine (NVVG). These one hundred fifty-five SIPs had earlier pronounced their commitment to contribute to the development of social insurance medicine. They had volunteered to participate in pro deo projects of their association to professionalise their work. All were working in disability evaluation for the Dutch Act on Insurance of Incapacity for Work (WAO).

### Protocols

In the Netherlands, three protocols to perform disability assessment interviews have been published, all based on practical experience: the Interview of Methodical Assessment (IMA: [24]), the Disability Assessment Structured Interview (DASI: [25]) and the Multi Causal Analysis [26]. Boer et. al. [27] report on a comparison of the protocols. For a detailed description of the protocols, see Additional file 1.

The protocols all describe semi-structured interviews, indicating the topics that need to be addressed during the interviews and their sequence. To a varying extent, the protocols describe the techniques or procedures of the interview such as the introduction, summaries and ending. All protocols are based on the principle of assessment on arguments [28], which means that the opinion of the claimant of his capacity and incapacity and his arguments for that opinion are to be discussed, completed if necessary and verified. This verification first takes place in the interview itself by comparing the claimant's opinion with other information such as facts regarding the past and future and his experiences other than in work. Furthermore, the SIP considers medical records, physical examinations, the history of sick leave, and return to work activities in order to form his opinion on the claimant's capacities. Finally, all protocols pay attention to the special context of social insurance, which makes the interviews different from medical examinations in health care [3,9,10,28]. The protocols prescribe a critical attitude for the SIPs and suggest special attention for the introduction to the interview in which a clarification of the purpose and procedure is explained to the claimant. The protocols do not describe conditions for interviewing such as time, the qualifications of SIPs or an optimal moment of assessment. The topics that address a claimant's disability can be compared to ICF [29] and a biopsychosocial approach [3], and can be said to match both. See Table 1 for this comparison.

**Table 1:** Interview protocols according to ICF and biopsychosocial approach (BPS)

ICF	BPS	IMA	DASI	MCA
Disease	Bio.	Health complaints, cause of disability	Information on disease.	Health and disease.
Impairments.	Bio.	Health complaints that prevent claimant from working. General health.	Information on disease.	Health and disease.
Activity limitations.	Bio.	Health complaints that prevent claimant from working. Activities of daily living.	Actual functioning.	Actual functioning.
Participation problems.	Social.	Claimant's perception of his capacity for own work. Claimant's perception of his capacity for other work.	Claimant's perception of his capacity to do his own or other work. Actual problems of participation.	Actual functioning.
Personal factors.	Psycho.	Motivations.	Perceived burden in the work.	Person.
Environmental factors.	Social.	Work description.	Work description.	Work description. Private situation.

The protocols show differences as well. The IMA provides the most detailed description of twelve topics in a fairly strict sequence. The DASI is less strict and uses the Listing of Functional Capacities (LFC) as a checklist, together with six other topics in a preferred sequence. The LFC is the output form in use at the Dutch Institute of Social Insurance and indicates six clusters of activities that are relevant for functioning in work. In the DASI, the claimant is asked to give examples of his actual functioning. The MCA is the least strict, providing five areas of conversation that need to be explored in a preferred sequence.

The topics of the different protocols resemble each other but are not precisely the same. The topics are partly medical such as 'Medical history' or 'General health', but also psychosocial such as 'Private situation', 'Motivation' or 'Life events'. Topics cover the experiences and events of the past, examples of which are 'Medical history' and 'Life events', and the present such as 'Claimant's opinion of his actual capacity for work' and expectations for the future.

The IMA invites the claimant to follow precisely the questions asked and not to elaborate on personal associations. Summaries in IMA are not only used as an interview technique but also as formal stepping stones for the conclusion. The DASI invites the claimant to describe his functioning with actual examples from everyday life. Summaries are used as an interview technique. The MCA strives to achieve maximal trust from the claimant by quickly focussing on the aspects that bother the claimant. Thus, it is expected that the claimant will open up and present his capacities and incapacities in an honest manner. Summaries are used as an interview technique – they are utilised to encourage the claimant's participation by showing that the SIP understands what the claimant says.

### Procedure and set-up

The authors formulated a list of questions on four subjects to investigate the research question cited above. The description of the protocols was used to draft the questionnaire. The questionnaire was mailed to the participating SIPs.

I. The first subject was the familiarity and the use of the protocols by the SIPs. The respondents were asked if they used one or more of the three protocols and if they had been trained in these. The answer could also be that they did the assessment their own way, not using any of the protocols.

II. The second subject was the direction of the interview in the situation of social insurance. The respondents were asked who decided on the topics of the interview and their sequence. The answers could be that the interview was structured, that there was an application of a sequence of topics, that the SIP or claimant determined the topics, and whether specific examples of limitations of activities were asked. The answers were categorised over the three protocols, a combination of these protocols, or labelled as 'own protocol'.

III. The third subject was the position of the claimant towards the SIP. Respondents were asked (1) if they always checked the information provided by the claimant, and (2) if having a good relationship with the patient during the assessment is important. The answers were categorised over the three protocols, a combination of these protocols, or labelled as 'own protocol'.

IV. The fourth question was about the topics that the SIPs basically always address during the disability assessment. A list of topics was proposed, based on the protocols. The answers were categorised over the three protocols, a combination of these protocols, or labelled as 'own protocol'.

### Data analyses

The number of participating SIPs, mean age, and years of experience were noted. The application of a protocol and having been trained in it were noted in percentages of the SIPs. The answers to the second and third questions were noted in percentages of SIPs, in total and per protocol. The answers to the fourth question were noted in the frequency of topics that are basically always addressed, in total and by protocol. Differences between the groups of SIPs concerning questions 1, 2 and 4 were tested using T-tests. A p-value < 0.05 was considered to be statistically significant. The answers to question 3 were analysed using the exact two-sided McNemar test, considering a p-value < 0.05 to be statistically significant.

### Ethics committee

This study was not submitted for ethical approval. The study includes physicians who are not asked to perform specific professional actions for this study but to fill in an anonymous questionnaire.

## Results

Of the hundred and fifty-five SIPs, ninety-eight returned a completed questionnaire (64%). Sixty-four SIPs (64% of 98 respondents) were male and the average age was 47.7 years (SD = 6.9). Sixty-six had more than 10 years' experience in disability evaluation based on the Dutch Act on Insurance of Incapacity for Work (WAO). We have no information on non-respondents.

Respondents were asked if they were trained in one or more of the three protocols and if they used them. Eighty-seven percent of the respondents were trained in IMA, forty percent in DASI and twenty-seven percent in MCA. All respondents used some form of protocol: twenty-three percent reported to use IMA, twelve percent DASI and twenty-two percent MCA, whilst forty-two percent reported to have constructed their own mix. We found no significant relationship between the training received and the use of a particular protocol.

Respondents were asked who determined the topics of the interview – the claimant or the SIP – and, if applicable, in what sequence. The results are shown in Table 2.

**Table 2:** Direction of the interview in total and by use of protocol, % yes

	**• Total**	'use IMA'	'use DASI'	'use MCA'	'use several'	'use own model'
***N:***	99	23	12	22	20	22
**%:**	(100%)	(23%)	(12%)	(22%)	(20%)	(22%)
**Interview follows a fixed pattern (N = 97)**	90%	95%	100%	77% ▼	95%	86%
**Use fixed sequence of items (N = 99)**	63%	70%	75%	50%	70%	55%
**Items are determined by claimant (N = 99)**	0%	0%	0%	0%	0%	0%
**Items first by claimant then by SIP (N = 99)**	39%	22% ▼	42%	50%	45%	41%
**Ask specific examples of limitations of activities (N = 99)**	75%	65%	75%	86%	80%	68%

Percentages are column percentages and are tested with the Pearson Chi-square test. The contrast is: 'subgroup' vs. 'other cases'. ▲ and ▼: p < 0.05 for significantly high and low percentages. Symbols are based on significance only, not on Effect Size. Tests and symbols refer to horizontal comparisons.

Ninety percent of the SIPs have their interview structured and sixty-three percent of the SIPs structure by applying a fixed sequence of topics as prescribed by the protocols. The others maintain structure on a more abstract level than on topics, indicating fields of discussion such as 'Private situation' or 'Person'. With none of the respondents the topics were determined by the claimant, but for thirty-nine percent of the SIPs, the claimant may have some room for his own topics at the start of the interview, after which the SIP takes over. Asking for specific examples of limitations of activities is done by seventy-five percent of the SIPs. The use of interview protocols affects only two aspects: a fixed pattern is less reported by users of MCA and leaving room for the claimant to start with his own topics is less seen with IMA. This is in agreement with these protocols.

SIPs were asked about their professional attitude towards the interviews. There are significantly more SIPs who recognise their role in having to verify what the claimant says (95%), than there are SIPs who recognise the need of establishing a good relationship (83%, p < 0.02, McNemar's test). Between users of a particular protocol, there are no significant differences.

SIPs were asked if the introduction to the interviews has a specific function. The results are shown in Table 3.

**Table 3:** Attitude towards the interview in total and by use of protocol, % yes

	**• Total**	'use IMA '	'use DASI'	'use mca'	'use several '	'use own model'
***N:***	97	22	12	22	19	22
**%:**	(100%)	(23%)	(12%)	(23%)	(20%)	(23%)
**Need to put the client at ease (N = 96)**	77%	57% ▼	50% ▼	91%	89%	86%
**Need to clarify the interview purpose (N = 97)**	94%	100%	100%	86%	84%	100%
**Need to clarify the interview procedure (N = 96)**	61%	81% ▲	50%	50%	68%	55%

Percentages are column percentages and are tested with the Pearson Chi-square test. The contrast is: 'subgroup' vs. 'other cases'. ▲ and ▼: p < 0,05 for significantly high and low percentages. Symbols are based on significance only, not on Effect Size. Tests and symbols refer to horizontal comparisons.

Clarifying the purpose of the interview is common amongst ninety-four percent of the respondents. The need to put the client at ease is recognised by seventy-seven percent and significantly less so by users of IMA and DASI. Users of MCA, of a combination of protocols and of their own protocol try to break the ice significantly more than those using IMA and DASI exclusively. Users of IMA were most keen on instructing the claimant about the procedure of the assessment with eighty-one percent, which is significantly higher than the sixty-one percent of the whole group.

Respondents were asked to name the topics they basically always address. These are shown in Table 4.

**Table 4:** Topics that are addressed in total and by use of protocol, % yes

		'use IMA'	'use DASI'	'use MCA'	'use several'	'use own model'
***N:***	**• Total 99**	23	12	22	20	22
**%:**	(100%)	(23%)	(12%)	(22%)	(20%)	(22%)
**Claimant's opinion fit for own work or other (N = 99)**	100%	100%	100%	100%	100%	100%
**Claim and health arguments (N = 99)**	99%	100%	100%	100%	95% ▼	100%
**Work (N = 99)**	99%	100%	100%	95%	100%	100%
**Perceived limitations of activities and obstacles (N = 99)**	97%	91%	100%	100%	100%	95%
**Actual functioning (N = 99)**	94%	96%	100%	91%	100%	86%
**Medical history (N = 99)**	95%	96%	100%	91%	100%	91%
**Private situation (N = 99)**	93%	91%	100%	95%	80% ▼	100%
**Activities/handicaps (N = 99)**	91%	87%	83%	91%	100%	91%
**Conclusion SIP (N = 99)**	90%	100%	100%	86%	90%	77% ▼
**Future (N = 99)**	88%	83%	92%	82%	95%	91%
**General Health (N = 99)**	82%	87%	92%	77%	95%	64% ▼
**Possibility to do other work (N = 99)**	86%	87%	83%	82%	90%	86%
						
**Motivation (N = 99)**	68%	70%	50%	77%	70%	64%
**Life-events (N = 99)**	67%	65%	58%	68%	70%	68%
**Change mentally, as a person (N = 99)**	62%	70%	58%	64%	70%	45%
**Person (N = 99)**	59%	52%	33%	68%	80% ▲	50%
**General health (N = 99)**	49%	43%	50%	55%	70% ▲	32%
**Causes of disability (N = 99)**	44%	52%	17% ▼	41%	65% ▲	36%

Percentages are column percentages and are tested with the Pearson Chi-square test. The contrast is: 'subgroup' vs. 'other cases'. ▲ and ▼: p < 0,05 for significantly high and low percentages. Symbols are based on significance only, not on Effect Size. Tests and symbols refer to horizontal comparisons.

Twelve topics are mentioned by over eighty percent of the respondents and six topics by between forty-four and eighty percent of the respondents. The claimant's opinion of his being fit for his own work or other work and his claim of incapacity and the health arguments he has for that claim stand at hundred percent. A description of the claimant's previous work reaches ninety-nine percent. The claimant's opinion of the 'causes of his disability' and his 'general health' do not reach an agreement of fifty percent of the respondents.

## Discussion

In this study, we examined the extent to which three Dutch interview protocols for the assessment of incapacity for work and their underlying principles were known and adhered to. The respondents were a selected group of experienced SIPs who were all doing assessments for the Dutch Act on Insurance of Incapacity for Work (WAO) in the Netherlands and motivated for professional development (N = 155).

### Main findings

Ninety-eight SIPs responded to the questionnaire. They were all trained in at least one of the protocols. Fifty-eight percent reported to use one of these and forty-two percent had constructed their own protocol. We found no significant relation between being trained in a protocol and using it. This corresponds with the finding that a single element of training without control on implementation does not yield stable results [30-32]. The results also indicate that SIPs do make their own mix of recommendations that are given by the different protocols. Respondents agreed on the idea of conducting a semi-structured interview, most often by using a fixed sequence of predefined topics. The protocols define eighteen topics altogether, twelve of which are basically always addressed by over 80% of the respondents. The SIPs recognised their position of having to verify what the claimant says and to make an effort to get good cooperation with the claimant rather than establishing a good relationship. Semi-structured interviews can lead to a more reliable gathering of information by using a construct of what is being assessed [23]. All protocols, although using loosely defined topics, can be said to use an implicit concept of disability. This concept matches the ICF and the biopsychosocial approach, both being recognised as authoritative in this field. All protocols aim at determining not only limitations but also capacities, which is in accordance with modern opinions about the participation of people with disabilities [33].

The context of assessment in social insurance implies the need for a fair trial and a critical attitude of the SIP [19]. A fair trial requires among others that the claimant must be invited to state his claim and his arguments. It is a professional choice to assess on the basis of this claim and arguments rather than to determine disability only on presumed objective medical findings. It is unsure however, how valid and reliable a claimant's opinion of his situation is and how he reports this during claim assessment [34-36].

### Strengths and weaknesses

This study reports the expert opinion of SIPs whose daily work it is to conduct interviews for the assessment of incapacity for work. The SIPs are not representative of all SIPs as they are selected on their ambition to contribute to their profession. With regard to adherence to the protocols they are probably a positive selection. The SIPs were all trained in one or more of the protocols and had had the opportunity to develop a protocol that served their daily needs. We asked the SIPs for their opinions on principles of interviewing in assessment of incapacity for work but we do not know how they perform in practice. It is uncertain to what extent protocolled interviews address ICF fields in an even manner in practice. Slebus et. al. [37] and Brage et.al [38] found that in assessment of incapacity for work personal factors and environmental factors were less addressed than the other fields of ICF.

### Impact

Our results open up a new way of quality control of the assessments by using a protocol to conduct the interviews. As the basic principles are accepted by the majority of SIPs their application can be assessed. It is possible to repeat this study in other countries to find the common principles that SIPs apply in different arrangements. That may make it possible to develop interview protocols elsewhere too. It seems likely that interview protocols need to be tailored to the arrangement at hand. Long term incapacity for work may need a different protocol from short term incapacity or for allowances for other handicaps. In any case further scientific testing is needed to establish more than face validity. The degree to which interviews in assessment of incapacity for work would best be structured is not known. Full structuring is not likely to be possible as many topics may be relevant in a specific case but there is no evidence to decide on what topics are the most relevant in all cases [39-41]. In order for such protocols to be effective they need to be implemented and applied in practice. Our study shows that earlier protocols were not blindly followed after training and we did not study why this was the case. It needs to be proved that a protocol that parts from accepted basic principles will do better. Some form of follow up after training will probably be necessary [31].

## Conclusion

One way of dealing with the susceptibility to bias of assessment interviews is to use protocols for interviewing the claimants. In Dutch practice several such protocols have been developed. These protocols correspond with concepts in the ICF and the biopsychosocial approach. Our study indicates that there is professional consensus among experienced Dutch SIPs about the principle of assessment on arguments, the principle of conducting a semi-structured interview and the most crucial interview topics. Crucial topics cover all fields of ICF. This consensus can, without striving for a detailed and universally applicable protocol, be used to further develop professional consensus on SIPs attitude, structuring of the interview and the selection of relevant topics that are more precisely circumscribed and based on evidence about what constitutes disability. This consensus can provide a starting point for further validation and development of a new protocol that can be implemented in practice and evaluated. It would need more than a single training in order to really be implemented. Some form of control is necessary.

If such a protocol is developed, implemented and controlled, the quality of the assessments would be improved in terms of transparency and reproducibility. The assessments would also become more comparable which would make them more accessible to scientific research on behaviour of both the SIP and the claimant. It would also enable claimants to better prepare themselves to the assessments which would make their position more equal to that of the SIP. The transparency of the reports and the satisfaction of the claimants would be endorsed by this.

## Competing interests

The authors declare that they have no competing interests.

## Authors' contributions

WELdB designed the study, did the field work and prepared the manuscript. HW participated in drafting the manuscript. FJHvD and HHBMW supervised the project and participated in drafting and revising the manuscript.

## Pre-publication history

The pre-publication history for this paper can be accessed here:



## Supplementary Material

Additional file 1 **Disability Interview Protocols**. The description of three disability interview protocols, procedure and topics. Click here for file

## References

[B1] Council of Europe (2002). Assessing disability in Europe. Strasbourg.

[B2] Gordon G (1966). Role theory and illness.

[B3] Waddell G, Aylward M (2005). The scientific and conceptual basis of incapacity benefits.

[B4] Mabbet D, Bolderson H, Hvinden B (2002). Definitions of disability in Europe: a comparative analysis.

[B5] de Boer WEL, Besseling JJM, Willems JHBM (2007). Organisation of disability evaluation in 15 countries. Pratiques et organisation des soins.

[B6] Health council of the Netherlands (2007). General introduction to the protocols for insurance medicine. [report in Dutch]. Den Haag.

[B7] Legner R, Cibis W (2007). Quality control in socio-medical assessment. [article in German]. Rehabilitation.

[B8] Becker E, Horn S, Hussla B, Irle H, Knorr I, Korsukewitz C, Pottins I, Rohwetter M, Schuhknecht P, Timner K (2003). Guidelines for the sociomedical assessment of performance in patients suffering from discopathy or associated diseases. [article in German]. Gesundheitswesen.

[B9] (2001). Bundesversicherungsanstalt für Angestellte, Landesversicherungsanstalten, Bundesknappschaft, Bahnversicherungsanstalt und Seekasse im Verband Deutscher Rentenversicherungsträger Medical assessment for legal disability insurance. [report in German].

[B10] Riemer Kafka G, (ed) (2007). Assessment in social insurance medicine. [report in German]. Schweizerische Ärzteverlag AG Basel.

[B11] Reck R (2005). Chronic back pain and expert opinion. [article in German]. Versicherungsmedizin.

[B12] Ydreborg B, Ekberg K, Nilsson K (2007). Swedish social insurance officers' experiences of difficulties in assessing applications for disability pensions – an interview study. BMC Public Health.

[B13] de Bont AA, Berendsen L, Boonk MPA, Brink JC van den (2000). At the social insurance physician's office.[report in Dutch]. CTSV Zoetermeer.

[B14] Chang YC, ChenSea MJ, Jang Y, Wang JD (2000). A simple self-rating assessment model of residual work capability for occupational permanent disabilities. American journal of industrial medicine.

[B15] Fleten N, Johnsen R, Forde OH (2004). Length of sick leave – why not ask the sick listed?. BMC public health.

[B16] Norrby E, Lindahl I (2006). Reliability of the instrument DOA: dialogue about ability related to work. Work.

[B17] Mendelson G, Mendelson D (2004). Malingering in the medicolegal context. Clin J Pain.

[B18] Bianchini KJ, Greve KW, Glynn G (2005). On the diagnosis of malingered pain-related disability: lessons from cognitive malingering research. Spine.

[B19] Aylward M, Halligan PW, Bass C, Oakly DA (2003). Origins, practice and limitations of disability assessment medicine. Malingering and illness deception.

[B20] Kerstholt J, de Boer WEL, Jansen NJ (2006). Disability assessments: effects of response mode and experience. Disabil Rehabil.

[B21] Brouwer S, Dijkstra PU, Stewart RE, Goeken LN, Groothoff JW, Geertzen JH (2005). Comparing self-report, clinical examination and functional testing in the assessment of work-related limitations in patients with chronic low back pain. Disabil Rehabil.

[B22] Spanjer J, Krol B, Brouwer S, Groothoff JW Interrater reliability in disability assessment base don a semi-structured interview report. Disabil Rehabil.

[B23] Conway JM, Jako RA, Goodman DF (1995). A meta-analysis of interrater and internal consistency reliability of selection interviews. J Appl Psych.

[B24] de Boer WEL, Duin JA, Herngreen H (1997). Manual for interviewing in methodic assessment [report in Dutch]. SSG Utrecht.

[B25] Spanjer J (2000). Capacity oriented assessment. [report in Dutch]. Amsterdam Lisv.

[B26] de Boer WEL, Meijers JM, Verhoeff M (2000). Multicausal analysis: theory and practice [report in Dutch].

[B27] de Boer WEL, Spanjer J, Wijers JHL (2006). Interview protocols in social Insurance medicine [article in Dutch]. TBV.

[B28] Health Council of the Netherlands (2005). Assessment, treatment and coaching [report in Dutch]. Den Haag.

[B29] World Health Organisation (WHO) (2001). International Classification of Functioning, Disability and Health (ICF).

[B30] Kok R, Hoving JL, JHAM Verbeek, Schaafsma FG, Smits PBA, Vlek FJM, Duin JH, van Dijk FJH (2008). Evidence based Insurance medicine: evaluation of a workshop on EBM. [article in Dutch]. TBV.

[B31] O'Brien MA, Rogers S, Jamtvedt G, Oxman AD, Odgaard-Jensen J, Kristoffersen DT, Forsetlund L, Bainbridge D, Freemantle N, Davis D, Haynes RB, Harvey E (2007). Educational outreach visits: effects on professional practice and health care outcomes. Cochrane Database of Systematic Reviews.

[B32] Davis D (1998). Does CME work? An analysis of the effect of educational activities on physician performance or health care outcomes. [Review]. Int J Psychiatry Med.

[B33] Organisation for Economic Co-operation and Development (OECD) (2003). Transforming Disability into Ability. Policies to Promote Work and Income Security for Disabled People. Paris.

[B34] Brouwer S, Dijkstra PU, Stewart RE, Goeken LN, Groothoff JW, Geertzen JH (2005). Comparing self-report, clinical examination and functional testing in the assessment of work-related limitations in patients with chronic low back pain. Disabil Rehabil.

[B35] McCahon S, Strong J, Sharry R, Cramond T (2005). Self report and pain behaviour among patients with chronic pain. Clin J Pain.

[B36] Reneman MF, Jorritsma W, Schellekens JM, Goeken LN (2002). Concurrent validity of questionnaire and performance based disability measurement in patients with chronic nonspecific low back pain. J Occup Rehab.

[B37] Slebus FG, Sluiter JK, Kuijer PP, Willems JH, Frings-Dresen MH (2007). Work-ability evaluation: a piece of cake or a hard nut to crack?. Disability and Rehabilitation.

[B38] Brage S, Donceel P, Falez F (2008). Development of ICF core set for disability evaluation in social security. Disabil Rehabil.

[B39] Franche RL, Krause N (2002). Readiness for return to work following injury or illness. Journal of occupational rehabilitation.

[B40] Hunt DG, Zuberbier OA, Kozlowski AJ, Berkowitz J, Schultz IZ, Milner RA, Crook JM, Turk DC (2002). Are components of a comprehensive medical assessment predictive of work disability after an episode of occupational low back trouble?. Spine.

[B41] Nusselder WJ, Looman CW, Mackenbach JP (2005). Nondisease factors affected trajectories of disability in a prospective study. J Clin Epidemiol.

