# Supplementary material for: Interviews for the assessment of long-term incapacity for work: a study on adherence to protocols and principles
Source: BMC Public Health. 2009 Jun 2;9:169. doi: 10.1186/1471-2458-9-169 (PMC2698854; doi:10.1186/1471-2458-9-169)
Supplement: Additional file 1 — Disability Interview Protocols. The description of three disability interview protocols, procedure and topics. [file 1471-2458-9-169-S1.doc]

Interview of Methodical Assessment (IMA)

1 The IMA protocol describes ten topics that need to be addressed by at least one question and more if the SIP thinks the topic to be relevant to the case he is handling. The topics are clustered into topics that permit the claimant to state his claim and topics that permit the SIP to check on plausibility and consistency. The topics are best addressed in the sequence of the protocol and for the first three topics, this is mandatory. The description of the IMA contains many detailed instructions on how to ask specific questions and how to interpret answers. This enables the SIP to draft a complete picture of the claimant in his situation.

2 The IMA protocol requires a precise introduction, in which the aim and procedure of the assessment are explained and in which the SIP stresses that the claimant’s opinion of his actual situation is of great importance and that the opinion of other people (for example, the treating physician) and events of the past will be dealt with later on during the interview. The claimant is asked to agree with these rules. Thus, the SIP introduces rules for the interview that challenge the claimant to show his self-consciousness and autonomy. This enables the SIP to see if the claimant is able to follow these rules.

3 A physical examination, if necessary, is scheduled after the interview.

4 After each topic, a summary is given by the SIP and after the entire interview, a general summary is given. After each summary, the claimant is invited to comment on it. At the end, the SIP gives his provisional opinion and explains the further procedure.

1. Claim items**:**
   - Work description: *Would you please describe the work you used to do?*
   - Claimant’s perception of his capacity for own work: *Do you think you could do that work now, fully or partly? If not, what do you experience in your health that prevents you from doing it?*
   - Claimant’s perception of his capacity for other work: *Do you think you could do other work? What would that need to look like?*
2. Items to check:
   - Motivation: *How do/ did you like doing the work you used to do?*
   - Claimant’s perception of the cause of disease and handicap: *What do you think to be the cause of your being ill and disabled?*
   - General Health: *Were you generally healthy and fit before you became disabled?*
   - Changes (mental, personal**):** *Would you say you have changed as a person over the past period of sick leave?*
   - Life – events: *Did you experience important events in the years before you reported sick? Which?*
   - Claimant’s perception of the future: *What do you expect about your future health/ work situation?*
   - Activities of daily living: *Could you please describe an ordinary day, e.g. yesterday and indicate what you did, how you managed that and whom you met, in a chronological order?*
   - Physical Examination is scheduled at the end of the assessment.

3) Conclusion of the SIP, for the moment, is relative to the claimant’s opinion.

Disability Assessment Structured Interview (DASI)

1 The SIP is focused on the differences between the pre-morbid state and the actual state that indicates disease. Another key role is played by concrete and detailed examples that the claimant gives or is asked to give of every activity he performs and of the restriction of capacity that he claims to experience. This serves to reduce possible malingering or aggravation by the claimant. These examples are used to identify residual capacity to work. A semi-structured interview is conducted in which topics are fixed by the SIP but their sequence is free. All topics must be discussed, preferably in order of the protocol, but the SIP can decide to do otherwise. The description of DASI does not give examples of questions but considerations as to why and how the different topics are of importance. The purpose of this method is to reach a systematic assessment of what is to be assessed – the claimant’s capacity for work.

DASI has a strong structure; in particular, in topics 3 and 4 the SIP asks for concrete and detailed examples, which must be consistent and plausible.

Ask further information through others (treating physician, employer etc.).

2 In DASI the SIP explains the purpose of the assessment and the procedure. The SIP summarises the claimant’s record. Putting the client at ease, the SIP explains the aim of the assessment.

3 A physical examination is scheduled after the interview.

4 At the end, the SIP states clearly his opinion of the claimant’s capacities.

1. Work description and perceived burden in the work *(motivation and consistency).*
2. Medical history and information on disease: complaints, cause, treatment *(impairments).*
3. Claimant’s perception of (in-) capacity in examples, if needed, with help of LFC *(restrictions of activities).*
4. Actual functioning and problems of participation: current activities and relationships *(focus on capacities).*
5. Claimant’s perception of his capacity to do his own or other work *(claimant’s position in the assessment).*
6. Physical examination *(consistency and plausibility).*
7. Opinion of the SIP.

Multi Causal Analysis (MCA)

1 MCA is designed to help the SIP to determine the causes of restricted functioning and so to be able to give suggestions to promote a return to work. The approach is biopsychosocial and the disability is primarily conceived of as behaviour. The instruction describes general principles, fields of discussion and the relevance of these. The emphasis is put to the claimant’s motivation and hindrances he experiences. The psychological and social aspects are determined as well as medical aspects. All subjects must be discussed but the order is free.

2 The SIP briefly explains the procedure and gives a short summary of the patient’s records.

A dialogue should be reached fast. A relationship of trust of the claimant in the SIP is necessary. Consequently, the SIP tries to explore the claimant’s opinion on his situation. The SIP shows an attitude of empathy, respect and interest by continually asking questions and by taking subjective perceptions of the claimant into account. There is much room for the claimant to follow his line of thought and for the SIP to decide how he wants to conduct the interview, provided he pays attention to all five fields of the discussion. This leads to a light structuring of the interview. Precise questioning reveals the plausibility and consistency of the image that the claimant puts forward and how serious his incapacity is. The purpose of this method is to reach an understanding evaluation.

3 A physical examination is scheduled after the interview.

4 The SIP’s final conclusion is stated clearly to the claimant, who is invited to react to that. The SIP presents his conclusion about limitations in functioning, with room to discuss remarks from the claimant. Then, the SIP explains the further procedure.

1. Health and disease *(actual complaints, medical history, treatment and restrictions as experienced by claimant).*
2. Work description *(description and stressors).*
3. Private situation *(description and stressors).*
4. Actual functioning *(micro and meso, activities for the restoration of health and resumption of work).*
5. Person *(coping, locus of control etc.).*
6. Physical examination.
7. Conclusion of the SIP, plan of action, if relevant, and plan of evaluation, if relevant.
